# The Functions and Disorders of the Reproductive Organs in Childhood, Etc.

**Published:** 1871-10

**Authors:** 


The Functions and Disorders of the Reproductive Organs in Childhood, Youth, Adult Age, and Advanced Life, considered in their Physiological, Social, and Moral Relations. By William Acton, M. R. C. S. Third American, from the fifth London edition. Philadelphia: Lindsay & Blakiston, Publishers.

This standard work is already well and favorably known to the profession, through its former editions.

